# Clinical efficacy of botulinum toxin type A in the treatment of fasciitis pain: A systematic review and meta-analysis

**DOI:** 10.1097/MD.0000000000034461

**Published:** 2023-07-28

**Authors:** Tong-Tong Li, Zhong-Yuan Liu, Ling Xiong, Zhi-Wen Zhang

**Affiliations:** a Affiliated Hospital of Hubei University of Chinese Medicine, Wuhan, China; b Hubei Provincial Hospital of Traditional Chinese Medicine, Wuhan, China; c Hubei Provincial Institute of Traditional Chinese Medicine, Wuhan, China.

**Keywords:** botulinum toxin type A, lumbar back fasciitis, meta-analysis, neck and shoulder fasciitis, pain, plantar fasciitis, systematic review

## Abstract

The purpose of this meta-analysis was to assess the effectiveness of botulinum toxin type A (BoNT-A) in reducing pain associated with fasciitis. By synthesizing the findings from multiple studies, we aimed to provide a comprehensive evaluation of the current evidence regarding the efficacy of BoNT-A in the treatment of fasciitis pain.

**Methods:**

To identify studies for our report, we conducted electronic database searches of Embase, PubMed, Web of Science, and the Cochrane Library from their inception to November 20, 2022. We included only randomized controlled trials that examined the therapeutic effects of BoNT-A on fasciitis pain, with the primary outcome measure being the visual analog scale. We conducted statistical analyses using RevMan 5.4 software.

**Results:**

Our final meta-analysis comprised 14 randomized controlled trials involving 537 participants, with 271 patients in the BoNT-A group and 266 patients in the control group. The overall effectiveness of BoNT-A in reducing fasciitis pain was significant, with a mean difference (MD) in visual analog scale score of −2.59 (95% confidence interval [CI], −3.36, −1.82); *P* < .00001; *I*^2^ = 88%. Subgroup analysis revealed that BoNT-A was particularly effective in treating plantar fasciitis (MD = −3.34 [95% CI, −4.08, −2.78]; *P* < .00001; *I*^2^ = 75%), lumbar back fasciitis (MD = −2.17 [95% CI, −3.82, −0.52]; *P* = .001; *I*^2^ = 93%), and neck and shoulder fasciitis (MD = −1.49 [95% CI, −2.76, −0.22]; *P* = .02; *I*^2^ = 61%).

**Conclusion:**

BoNT-A has a significant analgesic effect on fasciitis pain. Therefore, BoNT-A presents a promising alternative treatment option for fasciitis (PROSPERO 2022: CRD42022382805).

## 1. Introduction

Fasciitis, also known as fibrositis, is a nonspecific inflammation that occurs between muscles and fascia, commonly found in muscle-rich areas such as the neck, shoulder, lower back, and soles of the feet.^[1]^ Studies have shown that the nociceptors in fascial tissue can directly transmit mechanical or chemical signals into nociceptive signals, and proprioceptors may transform into nociceptors under mechanical stimulation, which then convert into pain signals.^[2]^ Therefore, fasciitis is one of the main causes of localized nonspecific pain that affects most populations.^[3]^ It is estimated that about 2% to 8% of the global population are affected by fasciitis.^[4]^ Plantar fasciitis is the main cause of chronic heel pain, accounting for 11% to 15% of all people with foot symptoms.^[5]^ Patients with neck and shoulder, as well as lower back fasciitis, often present with chronic nonspecific neck pain, shoulder pain, and lower back pain,^[6]^ with symptoms such as pain, stiffness, restricted movement, and weakness.^[7]^ Epidemiological surveys have shown that the incidence of myofascial pain syndrome in nonspecific neck pain is 100%,^[8]^ the incidence of low back pain is about 12% to 65%,^[9]^ and the lifetime incidence of shoulder pain is about 66.7%.^[10]^ Although the pathogenesis of these patients is complex, studies have shown that releasing their myofascial tissue can effectively alleviate their pain symptoms.^[11,12]^ Currently, there are various treatment methods used in clinical practice to control fasciitis pain, including oral anti-inflammatory and analgesic drugs, stretching exercises, laser therapy, corticosteroid injections, extracorporeal shockwave therapy, and even surgical treatment.^[13]^

Botulinum toxin (BTX), a Gram-positive bacterium belonging to the *Clostridium* genus of anaerobic spore-forming bacteria, produces potent neurotoxins that cause botulism. BTX was initially discovered by Belgian scientist Van Ermengem in 1897. Subsequently, scientists further classified it into 7 subtypes (A–G).^[14]^ In the early 1980s, Canadian ophthalmologist Alan Scott Dresner first used BTX injections to alleviate eye tremors and strabismus.^[15]^ Since then, BTX has been widely applied in the fields of medicine and esthetics.^[16]^ Botulinum toxin type A (BoNT-A) has become one of the most popular toxin types due to its stability and strong binding, along with its excellent performance in clinical applications.^[17]^

BoNT-A has a wide range of applications, including neurological disorders,^[18]^ muscle diseases,^[19]^ urological conditions,^[20]^ ophthalmic diseases, maxillofacial disorders,^[21]^ and esthetic dermatology.^[22]^ BoNT-A was initially used to treat myofascial pain in the early 1990s. Doctors found that patients’ pain was relieved when using BoNT-A to treat spasmodic conditions such as facial spasms and blepharospasm.^[23]^ Subsequently, a study in 1994 reported that BoNT-A could reduce abnormal stress in the fascia by interrupting muscle contractions, thereby achieving therapeutic effects on cervical paraspinal and shoulder girdle muscles affected by fasciitis pain syndrome.^[24]^ Recent clinical evidence suggests that BoNT-A may be a treatment option for chronic pain and musculoskeletal injuries.^[25,26]^ However, despite the effectiveness of BoNT-A in relieving painful muscle spasms, its efficacy in treating fasciitis pain remains uncertain, and previous research findings lack consensus.

The objective of this systematic review and meta-analysis is to provide an up-to-date summary of prospective comparative studies, limited to randomized controlled trials (RCTs), evaluating the efficacy of BoNT-A in the treatment of myofascial pain.

## 2. Materials and methods

We conducted this systematic review and meta-analysis in accordance with the guidelines provided in the Preferred Reporting Items for Systematic Reviews and Meta-Analyzes statement^[27]^ and the Cochrane Collaboration recommendations.^[28]^ We prospectively registered the study protocol on the internationally recognized PROSPERO registration system (https://www.crd.york.ac.uk/PROSPERO/) to ensure transparency and reproducibility of the study design and methodology (PROSPERO 2022: CRD42022382805). Since all our analyses were based on previously published data, ethical approval was not required.

### 2.1. Eligibility criteria

The study followed the PRISMA guidelines (2021) with eligibility criteria defined according to population, intervention, control group, outcome, and study design: (1) Population: participants aged 18 or above with fasciitis pain in any of the following areas (neck, shoulder, lower back, foot, etc.); (2) Intervention: intramuscular or subcutaneous injections of BoNT-A; (3) Control group: the study should include a control group, which can consist of patients receiving placebo, conventional treatment, or alternative treatments; (4) Outcome: the primary outcome for assessing study results is the visual analog scale (VAS) score of patients before and after treatment. Secondary outcome include functional or disability scores specific to the different affected areas. (5) Study design: only randomized controlled trials published in academic journals will be included.

The exclusion criteria were as follows: (1) animal studies; (2) pain or functional score data were not reported; (3) study patients had previously received injections or surgery; (4) case reports, reviews, technical reports, and other non-randomized studies; and (5) articles were not reported in English.

### 2.2. Search strategy

To ensure a comprehensive search, the electronic databases PubMed, Embase, Cochrane Library, and Web of Science were searched from their establishment to November 20, 2022. The search strategy employed a combination of Medical Subject Heading terms and keywords related to “fasciitis,” “fasciopathy,” “chronic fasciitis,” “lumbar back fasciitis,” “neck and shoulder fasciitis,” “Plantar fasciitis,” “Botulinum toxin type A,” “BoNTA,” “BTX,” “BoNT-A,” and “BoNT.” The detailed search strategy is presented in Supplemental Material 1, Supplemental Digital Content 1, http://links.lww.com/MD/J356, and this strategy is applicable to each included electronic database.

### 2.3. Study selection process

Two authors conducted independent screenings of study titles and abstracts to determine if they met the established inclusion and exclusion criteria. The reference lists of relevant papers were also reviewed to identify any studies that may have been missed in the database searches. Any disagreements between the authors were planned to be resolved through consensus or with the consultation of the third-party expert.

### 2.4. Data extraction

Two independent authors extracted the following descriptive primary information from selected studies: first author; year of publication; country; study design; target population; study groups; number of participants in the intervention and control groups; male/female ratio; average age of patients; details of the interventions; follow-up; outcome measures. If the literature’s quantitative or qualitative information was incomplete, the original article’s first author or corresponding author was consulted by email and asked for the original data.

### 2.5. Quality assessment

Two researchers used the Cochrane Collaboration’s tool for assessing risk of bias 2.0 (RoB 2.0) and an improved Jadad scale to evaluate potential biases and the quality of included literature.^[29–31]^ The Cochrane tool assessed 5 domains: bias arising from the randomization process, bias due to deviations from intended interventions, bias due to missing outcome data, bias in outcome measurement, and bias in selection of reported results. For each criterion, the researchers evaluated whether it posed a “low risk of bias,” “some concerns,” or “high risk of bias.” The Jadad scale assessed the quality of studies based on their description of randomization, double blinding, allocation concealment, withdrawals, and dropouts. Each criterion received a score of 0, 1, or 2, for unclear or inadequately described methods, described but not blinded methods, or described and adequately blinded methods, respectively. The score also accounted for the reporting of reasons and numbers of withdrawals and dropouts. The resulting scale ranged from 0 to 7, with a score of 1 to 3 indicating low-quality literature and a score of 4 to 7 indicating high-quality literature.

### 2.6. Statistical analysis

We used Review Manager software (RevMan Version 5.4; Cochrane Collaboration Group, Copenhagen, Denmark) to perform a statistical analysis of outcome measures in the included studies. In the meta-analysis, for continuous variables, we obtained the mean, standard deviation (SD), and sample size of the observation group and control group separately for the VAS score after treatment follow-up, and converted them to the same unit. Specifically, we converted them to a standardized unit of cm. The mean and SD were then combined to represent the mean difference (MD) along with a 95% confidence interval (CI). As for the results of functional and disability assessment scales, they are indicated using the standardized mean difference (SMD).

To estimate the SD of the mean difference, we used the following formula^[32]^:


$$\text{SD}=\sqrt{{\text{SD}}_{1}^{2}+{\text{SD}}_{2}^{2}-2R\times {\text{SD}}_{1}\times {\text{SD}}_{2}},$$

where SD_1_ is the standard deviation before treatment and SD_2_ is the standard deviation after treatment, assuming a correlation coefficient (*R*) of 0.5.

Net change in measurement values (MD) can be calculated as the follow-up measurement value minus the baseline measurement value.

For cases reported with standard error of the mean, the SD can be calculated by


$$\text{SD}=\text{SEM}\times \sqrt{n},$$

where *n* represents the sample size.

When only reporting the range and median of study values, we use Hozo et al’s method^[33]^ to calculate the SD.


$$\text{SD}\approx \{\begin{array}{ll} \frac{1}{\sqrt{12}}{\left[{\left(b-a\right)}^{2}+\frac{\left(a-2m+b\right)}{4}\right]}^{1/2\;} & n\leq 15\\ \frac{b-a}{4} & 15<n\leq 70\\ \frac{b-a}{6} & n>70 \end{array},$$

where *a* represents the minimum value, *b* represents the maximum value, *m* represents the median, and *n* represents the sample size.

When numerical values are presented only in images, we utilize GetData 2.26 to extract the values from the images.

We employed Cochrane *Q* test to examine heterogeneity among the various studies, as well as quantitative evaluation as an objective measure of heterogeneity. Heterogeneity is deemed insignificant when it ranges from 0% to 25%, moderate when it ranges from 25% to 50%, and significant when it exceeds 50%. When *I*^2^ exceeds 50%, significant heterogeneity is present, and a random effects model is used. Conversely, when *I*^2^ is less than or equal to 50%, a fixed effects model is used. We utilized subgroup analysis to explore the reasons for the observed heterogeneity.

We utilized forest plots to visually represent the differences in results between the BoNT-A group and the control group in all included studies. We conducted sensitivity analyses through the stepwise removal of individual study data to evaluate the impact of missing data on overall results. Additionally, we employed funnel plots to identify potential publication bias among the included studies. To evaluate the objectivity of the funnel plots, we performed Beggs and Egger tests using Stata 16.0 software. The significance level for all statistical analyses in the article is set at *P* < .05.

## 3. Results

### 3.1. Study selection

**Figure 1.**
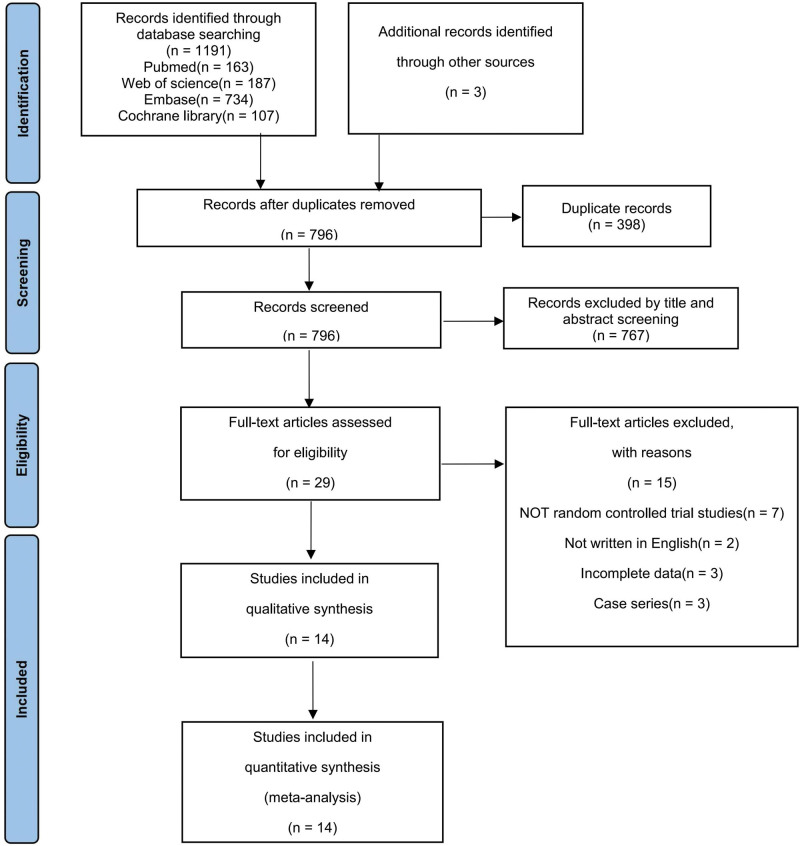
PRISMA (Preferred Reporting Items for Systematic Reviews and Meta-Analyzes) flow diagram of the study selection process. From the initial 1194 records, 14 studies were included.

Based on the aforementioned keywords, a preliminary search of 4 electronic databases yielded 1191 potentially relevant citations, including 163 from PubMed, 187 from Web of Science, 734 from Embase, and 107 from Cochrane Library. Three additional studies were obtained from other sources. After removing 398 duplicate records, 767 studies were excluded based on the assessment of their titles and abstracts. Following a thorough evaluation of the full text of the remaining 29 studies, 15 studies were excluded, leaving a total of 14 studies for inclusion in this meta-analysis.^[34–47]^ The process of study selection is illustrated in Figure 1.

### 3.2. Characteristics of included studies

The main characteristics of the 14 RCT studies screened above are shown in Table 1.

**Table 1:** Characteristics of the included studies

Author (year)	Country	Study design	Target population	Study groups	No. of patients	Male/female	Mean age	Injected dose (volume)	Follow-up (month)	Outcome measures
Braker et al (2007)^[34]^	Canada	Randomized double-blind, placebo-controlled trial	Neck shoulder fasciitis	BTX-A normal saline	10 10	6/4 6/4	48.0 ± 9.3 45.6 ± 10.7	50 U (1 mL) 1 mL	24 weeks	VAS, VRS, SF-36
Machado et al (2016)^[35]^	USA	Randomized double-blind, placebo-controlled trial	Lumbar back fasciitis	BTX-A Normal saline	18 19	14/4 10/9	51.3 48.6	500 U (6 mL) 6 mL	6 months	VAS, OLBPQ, ACPA
Lew et al (2008)^[36]^	USA	Randomized, double-blind, placebo-controlled pilot trial	Neck shoulder fasciitis	BTX-A normal saline	15 15	8/7 12/3	48.7 ± 9.4 48.5 ± 13.4	50 U (1 mL) 1 mL	6 months	VAS, NDI, SF-36
Huang et al (2010)^[37]^	China Taiwan	Randomized double-blind, placebo-controlled trial	Plantar fasciitis	BTX-A normal saline	25 25	6/19 6/19	54.4 ± 9.6 51.5 ± 5.5	50 U (1 mL) 1 mL	3 months	VAS, fat pad thickness
Ahmad et al (2016)^[38]^	USA	Randomized controlled trial	Plantar fasciitis	BTX-A normal saline	25 25	6/19 8/17	48.6 51.3	100 U (2 mL) 2 mL	12 months	VAS, FAAM
Ney et al (2004)^[39]^	USA	Prospective, randomized controlled trial	Lumbar back fasciitis	BTX-A normal saline	30 30	21/9 21/9	46.6	100 U (1 mL) 1 mL	6 months	VAS, OLBPQ, CLBPQ
Elizondo-Rodriguez et al (2013)^[40]^	Mexico	Randomized double-blind, placebo-controlled trial	Plantar fasciitis	BTX-A corticosteroid	19 17	10/9 6/11	41.6 44.5	250 U (4 mL) 2% lidocaine (2 mL) and 8 mg of dexamethasone (2 mL)	6 months	VAS, FADI, AOFAS
De Andrés et al (2010)^[41]^	Spain	Randomized double-blind, placebo-controlled trial	Lumbar back fasciitis	BTX-A Normal bupivacaine	27 27	7/20 7/20	NA NA	50 U (5 mL) 5 mL	3 months	VAS
Foster et al (2001)^[42]^	USA	Randomized double-blind, placebo-controlled trial	Lumbar back fasciitis	BTX-A normal saline	14 14	7/7 7/7	47.0 46.4	200 U (4 mL) 4 mL	8 weeks	VAS, OLBPQ
Babcock et al (2005)^[43]^	USA	Short-term, randomized, placebo-controlled double-blind trial	Plantar fasciitis	BTX-A normal saline	22 21	15/7 11/10	38.1 ± 5.9 38.2 ± 10.2	70 U (0.7 mL) 0.7 mL	8 weeks	VAS
Cogné et al (2017)^[44]^	France	Randomized, double-blinded crossover trial	Lumbar back fasciitis	BTX-A normal saline	9 8	3/6 0/8	38.1 ± 5.9 38.2 ± 10.2	200 U (4 mL) 4 mL	4 months	VAS, QBPDS
Abbasian et al (2019)^[45]^	Iran	Randomized double-blind, placebo-controlled trial	Plantar fasciitis	BTX-A normal saline	15 13	9/6 9/4	47.3 ± 6.1 45.6 ± 9.7	70 U (1.5 mL) 1.5 mL	12 months	VAS, AOFAS
Kwanchuay et al (2015)^[46]^	Thailand	Randomized double-blind, placebo-controlled trial	Neck shoulder fasciitis	BTX-A normal saline	24 24	4/20 2/22	39.8 ± 10.1 38.8 ± 10.8	20 U (0.2 mL) 0.2 mL	6 weeks	VAS, PPT
Ahadi et al (2022)^[47]^	Iran	Prospective, randomized controlled trial	Plantar fasciitis	BTX-A corticosteroid	17 18	6/11 2/16	47.2 ± 9.9 43.9 ± 8.6	150 U (1.5 mL) 1 mL of methylprednisolone plus 1 mL of normal saline	6 months	VAS, FAAM

Data are presented as mean ± SD.

ACPA = American Chronic Pain Association’s quality of life scale, AOFAS = American Orthopaedic Foot and Ankle Society, CLBPQ = Clinical Low Back Pain Questionnaire, FAAM = Foot and Ankle Ability Measures, FADI = Foot and Ankle Disability Index, NDI = Neck Disability Index, OLBPQ = Oswestry Low Back Pain Disability Questionnaire, PPT = pressure pain threshold, QBPDS = Quebec Back Pain Disability Scale, SD = standard deviation, VAS = visual analog scale, VRS = verbal rating scale.

The studies were conducted in 8 countries, including Canada,^[34]^ the USA,^[35,36,38,39,42,43]^ China,^[37]^ Mexico,^[40]^ Spain,^[41]^ France,^[44]^ Iran,^[45,46]^ and Thailand.^[47]^ The study population consisted of 537 patients diagnosed with fasciitis, with 242 patients having plantar fasciitis, 199 having lumbar back fasciitis, and 96 having neck and shoulder fasciitis. The average age of the patients ranged from 38.1 to 54.4 years, and there were more female patients than male patients (308/229). The patients were randomly assigned to either an experimental group that received BoNT-A injection treatment or a control group that received saline injection or corticosteroid^[40,47]^ injection treatment. Since the injection sites and dosages varied among studies due to the different locations of fasciitis, the results of all studies were evaluated using a VAS.

### 3.3. Risk of bias in studies

**Figure 2.**
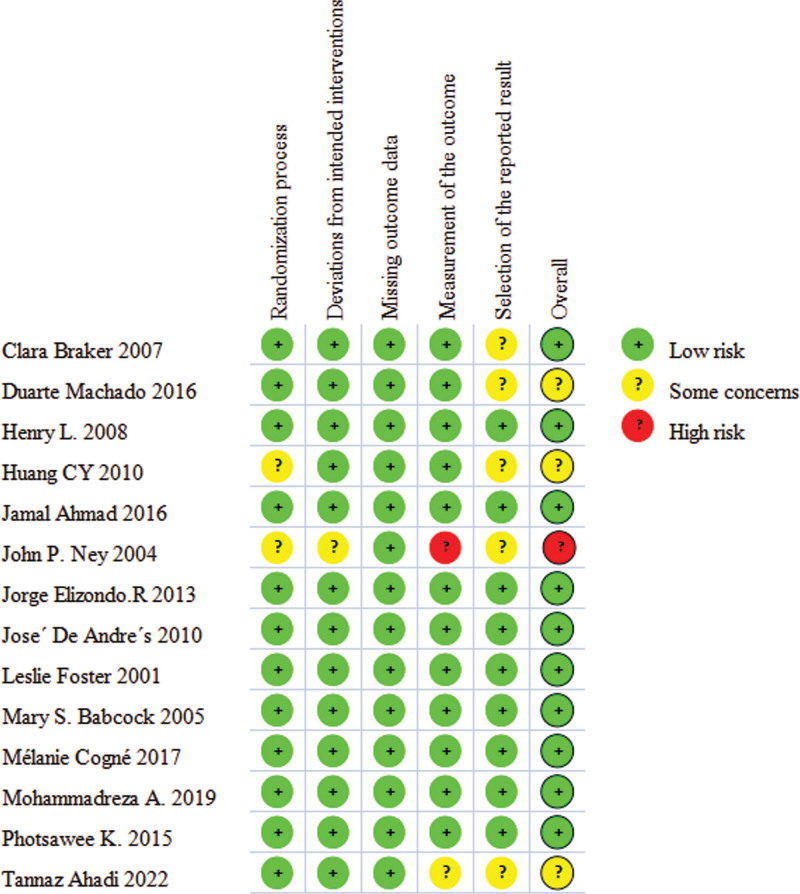
Risk of bias assessment of the studies included in this meta-analysis.

Figure 2 presents the results of the risk of bias assessment of the included studies using the Cochrane Collaboration Risk of Bias Tool 2.0. Of the 14 studies, 2 studies^[37,39]^ did not clearly explain their randomization process, resulting in having some concerns. All studies had low risk of bias for deviations from intended interventions, except for 1 study^[39]^ where the blinding was not explicitly described. In all the included studies, the risk of missing follow-up data on the final outcome was found to be low. The measurement of the outcome was some concerns in one study^[47]^ and high risk in another study.^[39]^ In 5 studies,^[34,35,37,39,47]^ the selection of the reported result was considered to have some concerns. Overall, the risk of bias in the evidence analyzed in our meta-analysis was identified as having some concerns^[35,37,47]^ with only one study^[39]^ having a high risk of bias. In addition, other studies were considered to have low risk.

### 3.4. Jadad quality assessment

Of the 14 randomized controlled trials included, 6 studies^[34,36,40,41,43,46]^ detailed the generation of the random sequence using a computer-generated random number or method. The remaining 8 studies^[35,37–39,42,44,45,47]^ only described the study as a randomized controlled trial. Among these studies, 6^[36,38,41–43,46]^ described the method of allocation concealment as central or pharmacy-controlled allocation or on-site computer control, sealed opaque envelopes, or other methods that prevent clinical doctors and subjects from predicting the assignment sequence. One study^[39]^ did not mention allocation concealment. The blinding method was mentioned in 8 studies^[36–38,41–44,46]^ which stated the use of completely identical placebo injections, while the remaining 6 studies only described the implementation of blinding during the study process. Ten studies^[34–36,39,40,42–45,47]^ described the reasons for withdrawal and loss to follow-up, while the remaining 4 studies did not. In the end, 9 studies^[34,36,38,40–44,46]^ were assessed as high-quality studies (score > 4), 4 studies^[35,37,45,47]^ were assessed as medium-quality studies (score = 4), and 1 study^[39]^ was assessed as low-quality (score < 4) (Table 2).

**Table 2:** Study design and quality rating.

Study	Described as randomized (0-2)	Allocation concealment (0–2)	Double blinding (0–2)	Withdrawals and dropouts (0–1)	Score
Braker et al (2007)^[34]^	2	1	1	1	5
Machado et al (2016)^[35]^	1	1	1	1	4
Lew et al (2008)^[36]^	2	2	2	1	7
Huang et al (2010)^[37]^	1	1	2	0	4
Ahmad et al (2016)^[38]^	1	2	2	0	5
Ney et al (2004)^[39]^	1	0	1	1	3
Elizondo-Rodriguez et al (2013)^[40]^	2	1	1	1	5
De Andrés et al (2010)^[41]^	2	2	2	0	6
Foster et al (2001)^[42]^	1	2	2	1	6
Babcock et al (2005)^[43]^	2	2	2	1	7
Cogné et al (2017)^[44]^	1	1	2	1	5
Abbasian et al (2019)^[45]^	1	1	1	1	4
Kwanchuay et al (2015)^[46]^	2	2	2	0	6
Ahadi et al (2022)^[47]^	1	1	1	1	4

### 3.5. Synthesis of results and meta-analysis

**Figure 3.**
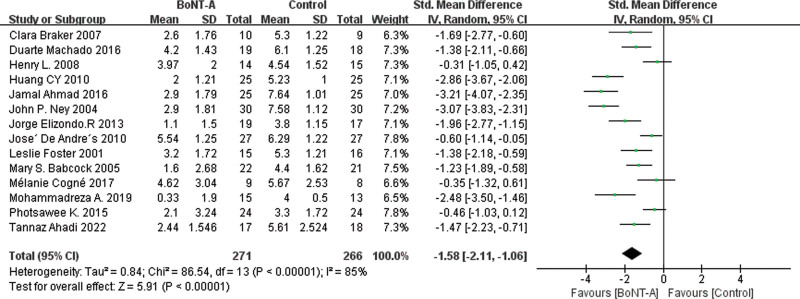
Forest plot for the comparison of the VAS for BoNT-A injections versus control groups. BoNT-A = botulinum toxin type A, VAS = visual analog scale.

**Figure 4.**
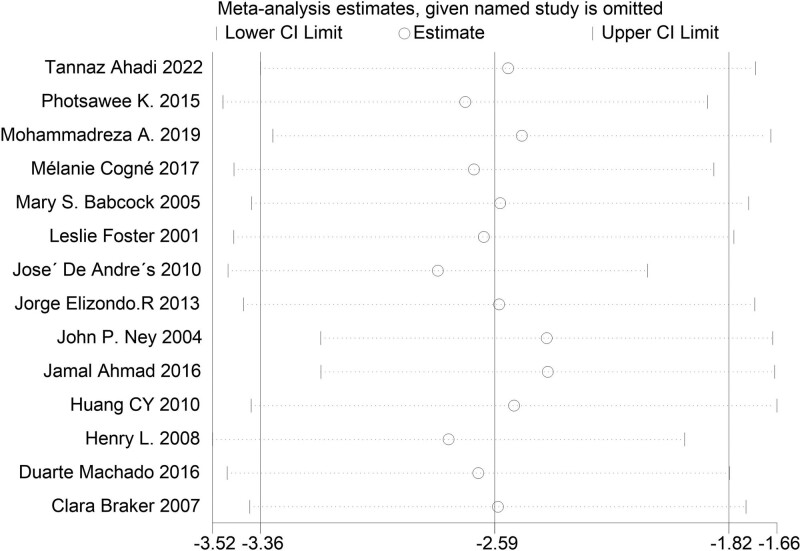
Influence analysis of pooled mean difference.

The main results of all the included studies were the changes to the VAS of pain after BoNT-A injection compared with the control groups. The results, as shown in Figure 3, revealed that the VAS score of the BoNT-A injection groups was significantly lower than that of the control groups (MD = −2.59 [95% CI, −3.36, −1.82]). In leave-one-out sensitivity analysis, the effect size remained stable upon removal of any individual study, indicating the robustness of the main results, as demonstrated in Figure 4 and Supplemental Material 2, Supplemental Digital Content 2, http://links.lww.com/MD/J357. Cochrane *Q* test and *I*^2^ quantitative analysis indicated significant heterogeneity (*P* < .00001, *I*^2^ = 88%). We employed subgroup analysis to explore the reasons for this heterogeneity.

**Figure 5.**
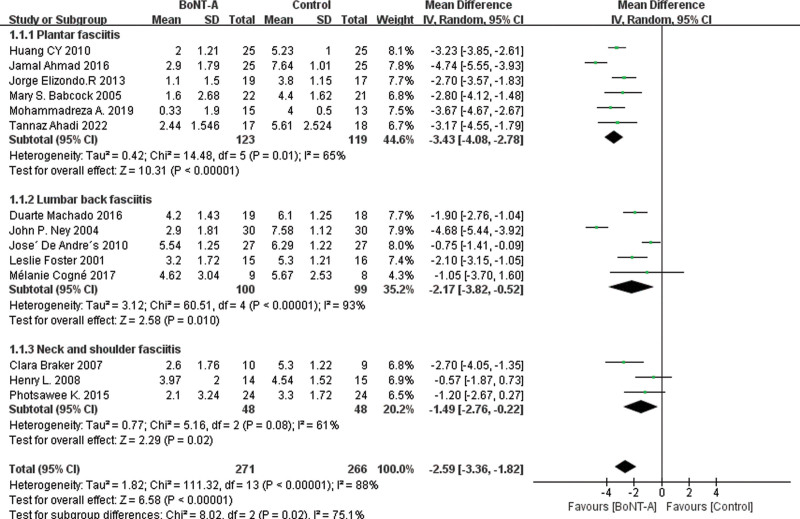
Forest plot showing VAS scores in subgroups of plantar fasciitis, lumbar back fasciitis, and neck shoulder fasciitis. VAS = visual analog scale.

The results of the forest plot grouped by the pathogenic location of fasciitis are displayed in Figure 5. Of the 14 studies, 6 reported plantar fasciitis,^[37,38,40,43,45,47]^ 5 reported lumbar back fasciitis,^[35,39,41,42,44]^ and 3 reported neck and shoulder fasciitis.^[34,36,46]^ The forest plot of the analysis results for each group is presented in Figure 4. We observed the most significant effect in the plantar fasciitis group (MD = −3.34 [95% CI, −4.08,−2.78]; *P* < .00001; *I*^2^ = 75%). The effect was second to none in the low back fasciitis group (MD = −2.17 [95% CI, −3.82, −0.52]; *P* = .001; *I*^2^ = 93%) compared to the neck and shoulder fasciitis group (MD = −1.49 [95% CI, −2.76, −0.22]; *P* = .02; *I*^2^ = 61%). This may be related to the number of included studies. The differences were statistically significant (*P* < .05).

**Figure 6.**
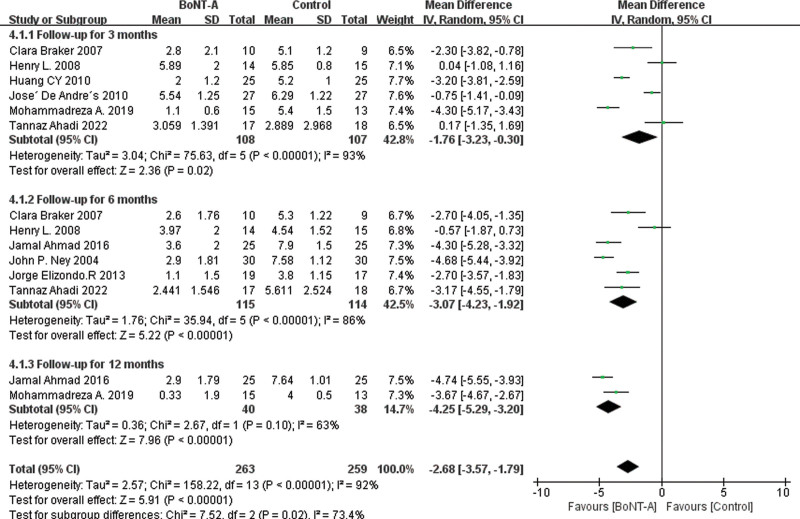
Forest plot for VAS scores during the follow-up period between the BoNT-A injection group and the control group. BoNT-A = botulinum toxin type A, VAS = visual analog scale.

The result of forest plot stratified by follow-up time after treatment is presented in Figure 6. Among the 14 studies, 6 studies^[34,36,37,41,45,47]^ reported VAS scores at 3 months after the end of treatment, 6 studies^[34,36,38–40,47]^ reported VAS scores at 6 months after treatment, and 2 studies^[38,45]^ reported VAS scores at 12 months after the end of treatment. The results of the meta-analysis demonstrated that at 12 months after treatment, the BoNT-A group showed the most significant improvement in VAS scores compared to the control group (MD = −4.25 [95% CI, −5.29, −1.70]; *P* < .00001, *I*^2^ = 63%). The second most significant improvement was observed at 6 months (MD = −3.07 [95% CI, −4.23, −1.92]; *P* < .00001, *I*^2^ = 86%). The improvement in VAS scores at 3 months was also significant (MD = −1.76 [95% CI, −3.23, −0.30]; *P* < .00001, *I*^2^ = 93%).

**Figure 7.**
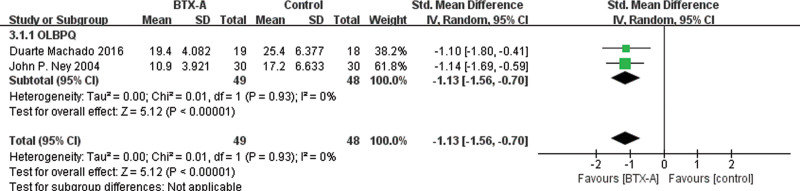
Forest plot for the comparison of the OLBPQ for BoNT-A injections versus control groups. BoNT-A = botulinum toxin type A, OLBPQ = Oswestry Low Back Pain Incapacity Questionnaire.

**Figure 8.**
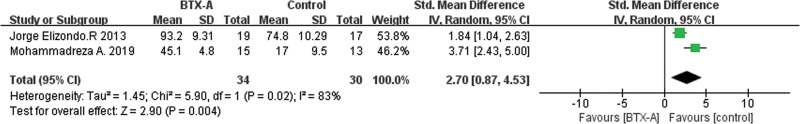
Forest plot for the comparison of the AOFAS for BoNT-A injections versus control groups. AOFAS = American Orthopedic Foot and Ankle Society, BoNT-A = botulinum toxin type A.

Given the variety of fasciitis sites covered in the included studies, we conducted additional analyses of function or disability improvement for specific sites, as a secondary outcome measure, in addition to VAS pain scores. In particular, 3 studies^[35,39,42]^ reported statistical scores of the Oswestry Low Back Pain Disability Questionnaire, with the meta-analysis results presented in Figure 7 (SMD = −1.13 [95% CI, −1.56, −0.70]; *P* < .00001; *I*^2^ = 0%), demonstrating a statistically significant difference. Furthermore, 2 studies^[40,45]^ reported the American Orthopedic Foot and Ankle Society (AOFAS) hindfoot-ankle score, and the meta-analysis result presented in Figure 8 showed a statistically significant difference (SMD = 2.70 [95% CI, 0.87, 4.53]; *P* = .004; *I*^2^ = 83%).

**Figure 9.**
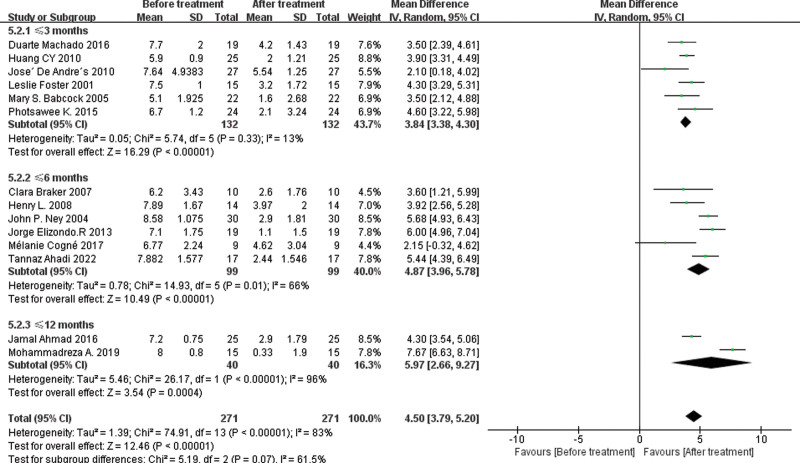
Forest plot displaying the mean difference and 95% CI for the effect of BoTN-A injections on pain (visual analog scale) at pre- and postinjection follow-up. BoNT-A = botulinum toxin type A, CI = confidence interval.

To compare with the minimum clinically important difference (MCID) for pain relief, we extracted the baseline and follow-up measurements of VAS score separately for the BoNT-A group. As shown in Figure 9, the treatment achieved statistically significant improvement in VAS score at ≤ 3 months (MD = 3.84 [95% CI, 3.38, 4.30]; *P* < .00001; *I*^2^ = 13%), ≤6 months (MD = 4.87 [95% CI, 3.96, 5.78]; *P* < .00001; *I*^2^ = 66%), and up to 12 months (MD = 5.97 [95% CI, 2.66, 9.27]; *P* < .00001; *I*^2^ = 13%). Based on the synthesis of previous studies,^[48–50]^ we conclude that the difference in the improvement of VAS score after treatment exceeds 2 cm, indicating the achievement of MCID. The meta-analysis results suggest that BTX injection can significantly improve pain score in patients with fasciitis, reaching the MCID level as early as the third month.

### 3.6. Sensitivity analysis and publication bias

**Figure 10.**
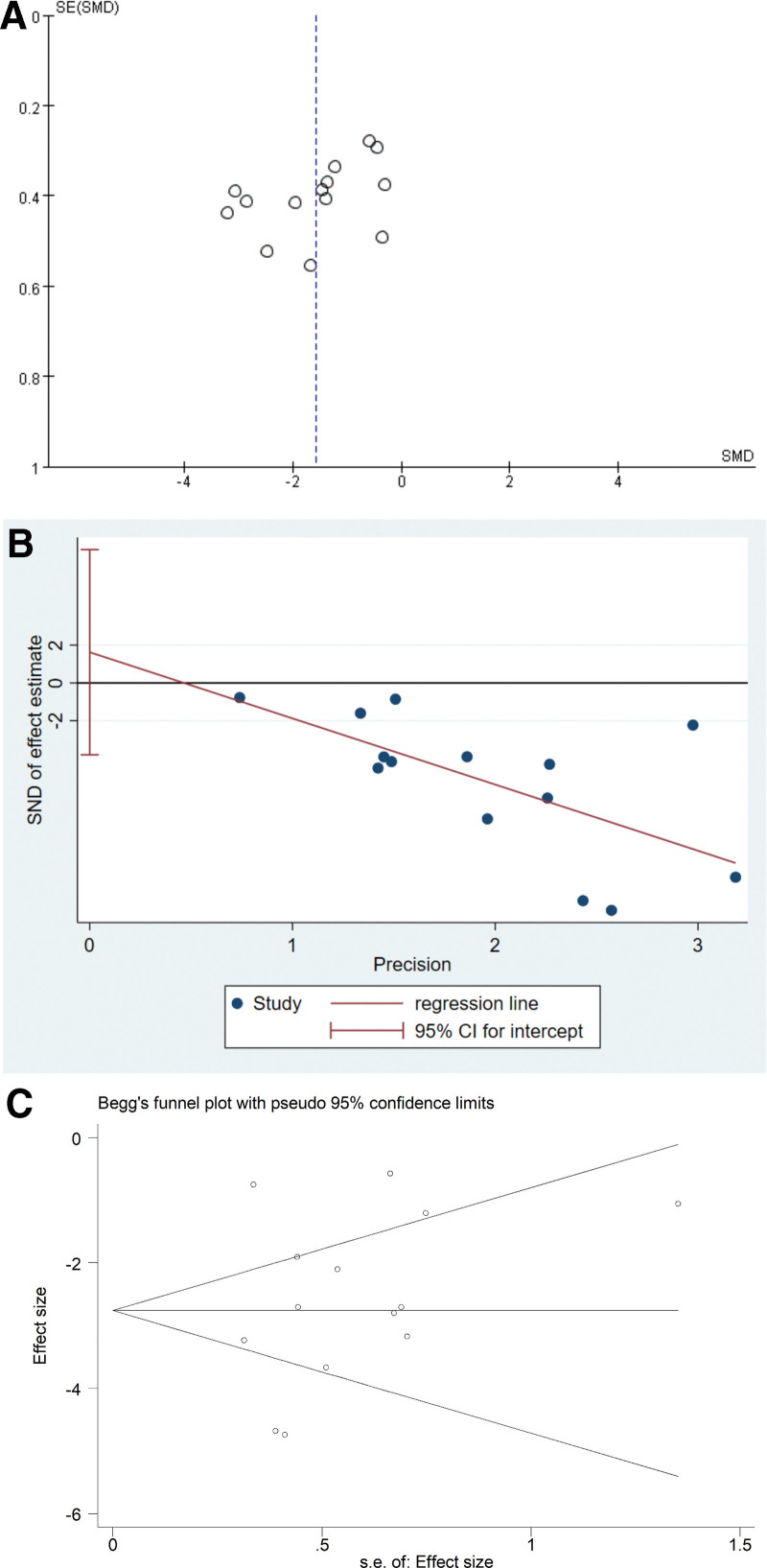
(A) Funnel plot assessing publication bias of the meta-analysis. (B) Egger funnel plot examining publication bias. (C) Begg funnel plot examining publication bias.

The funnel plot of all included studies, as shown in Figure 10A, is approximately symmetrical, indicating a low risk of publication bias. Additionally, both Egger test (*P* = .526, 95% CI [−3.8, 7.1]) shown in Figure 10B and Begg test (Pr > |*z*| = 0.381) presented in Figure 10C, did not indicate the presence of publication bias in the meta-analysis.

## 4. Discussion

The fascia is a connective tissue that tightly covers the surfaces of tissues and organs, providing remarkable toughness and elasticity that enable it to withstand mechanical stress and deformation.^[51]^ Recent studies have demonstrated that the fascia tissue plays a vital role in proprioception, that is, the sense of the body’s position and movement in space, force transmission, and injury perception.^[52]^ For example, fasciitis can cause various pain syndromes, including back pain, plantar fasciitis, periarthritis of the shoulder, and cervical pain, among others.^[3]^ Additionally, fasciitis is associated with various factors, such as direct injury, immune responses, weight-bearing, chronic strain, and abnormal stress on the musculoskeletal system.^[53]^ Moreover, fascial tissue has been proven to have active contractile properties,^[54]^ which can lead to mechanical imbalances in the musculoskeletal system. Therefore, the treatment of fasciitis should consider the interdependence between the fascia, muscles, and pain. The current treatment options for fasciitis encompass extracorporeal shockwave therapy, corticosteroid injections, nonsteroidal anti-inflammatory drugs,^[55]^ dry needling, and ultrasound therapy.^[56]^ Among these, extracorporeal shockwave therapy is primarily used for plantar fasciitis,^[57]^ while its application in cervical, shoulder, and lumbar fasciitis is less reported. Corticosteroid injections and oral nonsteroidal anti-inflammatory drugs offer prompt relief of pain symptoms but do not provide a cure for fasciitis; they only alleviate symptoms, and long-term usage may lead to dependence and drug resistance. Other physical therapy modalities such as dry needling, ultrasound therapy, laser therapy, and low-frequency pulse therapy^[58]^ have potential as treatment options for fasciitis, but their effectiveness is still being explored due to limited experimental studies and varying patient acceptance. In this study, we conducted a meta-analysis to assess the therapeutic efficacy of BoNT-A for fasciitis in the neck, shoulder, lumbar, and plantar regions.

Our study assessed the therapeutic efficacy of BoNT-A in relieving pain associated with 3 distinct sites of fasciitis, utilizing the VAS score as the primary outcome measure. To the best of our knowledge, this is the first comprehensive study to evaluate the effectiveness of BoNT-A treatment specifically for pain associated with fasciitis in these 3 different locations. The findings of the study indicate that BoNT-A exhibits superior efficacy in alleviating pain levels among patients with fasciitis when compared to treatment with saline or corticosteroids (MD = −2.59 [95% CI, −3.36, −1.82]; *P* < .00001). Sensitivity analysis further confirms the robustness of these results. Subgroup analysis, stratified by the site of fasciitis, indicated that the observed beneficial effect was most significant in patients with plantar fasciitis and lumbar fasciitis, while relatively less prominent in individuals with neck and shoulder fasciitis pain syndrome. This discrepancy may be attributed to the limited sample size in our study, emphasizing the need for more high-quality literature in the future to comprehensively evaluate the efficacy of BoNT-A in treating fasciitis across other body sites.

In the subgroup analysis conducted according to the follow-up period after treatment, we observed that with longer treatment durations, the BoNT-A injection group exhibited more sustained and significant improvement in VAS scores compared to the control group. Furthermore, out of the included studies, only 2^[34,45]^ reported adverse reactions occurring in a limited number of patients who received BoNT-A injections during the follow-up period. These adverse reactions included transient pain, local inflammation, and systemic symptoms such as weakness, fever, and dizziness. However, all adverse events were mild and temporary, and the researchers did not intervene, so they were not included in the statistical analysis.

To facilitate a more comprehensive comparison and evaluation of the efficacy of BoNT-A on fasciitis pain, we performed a separate analysis of the baseline and posttreatment VAS scores for all patients receiving BoNT-A across the included studies. This analysis involved comparing the scores to the predetermined MCID for pain relief. It is important to note that the control group received different injection treatments, including placebos or saline solutions. It is worth noting that there is currently no universally agreed-upon MCID for pain level relief in fasciitis. However, in studies specifically focusing on plantar fasciitis, an MCID level is considered to be achieved when the MD in VAS values before and after treatment exceeds 0.8 or 0.9 cm.^[50]^ Therefore, based on the previous MCID identified in patients with nonspecific neck pain and low back pain,^[48,49]^ we synthesized the applicable MCID for our study. Specifically, we defined the treatment effect as effective when the VAS score showed an improvement of more than 2 cm from baseline data. The findings from our meta-analysis demonstrated that the treatment outcomes of BoNT-A were not only effective but also statistically significant over time.

Notably, in addition to VAS scores, we analyzed alternative scales to evaluate the overall effectiveness of BTX treatment for fasciitis and improvements in the health status of patients. For instance, studies by Machado et al,^[35]^ Ney et al,^[39]^ and Foster et al^[42]^ included statistical scores from the Oswestry Low Back Pain Disability Questionnaire. Through additional subgroup analyses of these 3 studies, we found statistically significant differences. Elizondo-Rodriguez et al^[40]^ and Abbasian et al^[45]^ utilized the AOFAS ankle-hindfoot score in their study of plantar fasciitis. The results of the subgroup analysis indicated that the AOFAS score in the BoTN-A group was significantly better than that in the control group, with a statistically significant difference. Furthermore, 2 studies^[34,36]^ also conducted statistical analysis on the SF-36 quality of life questionnaire before and after treatment. Both studies reported higher SF-36 scores in patients treated with BoNT-A compared to the control group. Due to the limited number of articles, we were unable to perform subgroup analysis for all study metrics. Therefore, future studies with more extensive data are needed to enhance the accuracy of our findings. In summary, based on the results of the aforementioned analyses, it can be concluded that BoNT-A effectively alleviates fasciitis pain and improves functional impairment and quality of life in the affected area of patients.

BoNT-A is a subtype of BTX widely used due to its low serum toxicity and prolonged analgesic effect.^[59]^ Previous studies have shown that BoNT-A has the potential to relieve fascial or muscular-derived pain.^[60,61]^ BoNT-A has demonstrated superior efficacy over other similar analgesic agents, with a more sustained effect and fewer side effects after a single injection.^[62]^ BoNT-A’s therapeutic effects on fasciitis can be attributed to several mechanisms, including inhibition of presynaptic acetylcholine release, which can reduce muscle tension and indirectly relieve pain^[63]^; suppression of nociceptive neurotransmitter release, such as glutamate, substance P, and calcitonin gene-related peptide, in nerve pathways, producing analgesic effects^[64]^; and participation in the regulation of inflammatory substances, such as interleukins IL-18, IL-1β, and IL-10, produced by nerve damage, which can reduce inflammation and pain.^[65]^ These findings suggest that BoNT-A has significant potential in addressing the pathological processes involved in fascia-muscle-pain cycling, making it a promising therapeutic approach. Unfortunately, reviews and systematic evaluations of BoNT-A for the treatment of fasciitis remain scarce, and research conclusions are not consistent.^[66]^ In this study, we included the latest clinical RCT studies on the treatment of plantar fasciitis, neck, and lower back pain, evaluated BoNT-A’s therapeutic effects on 3 different sites of fasciitis, and summarized its powerful mechanisms. By improving previous research conclusions and broadening the research scope, we aim to promote the application of BoNT-A in the treatment of fasciitis pain.

## 5. Limitation

In this study, our objective was to conduct a comprehensive analysis of various aspects related to the use of BoNT-A in the treatment of fasciitis. However, we encountered certain research gaps and limitations throughout the study. First, the literature we included provided limited information regarding the occurrence of adverse reactions during BoNT-A treatment for fasciitis. Therefore, further studies are required to evaluate the long-term benefits and potential risks of repeated BTX treatments. Secondly, due to variations in efficacy indicators used across different literature, it was challenging to perform a meta-analysis encompassing all relevant factors. Additionally, there was a moderate to high level of heterogeneity observed in most of the studies, which was not addressed in the subgroup analysis. We believe that heterogeneity may have stemmed from various sources. First, the participants were from different countries, which could contribute to the heterogeneity of the study population. Second, different studies utilized diverse injection methods and sites, which might also contribute to heterogeneity. Lastly, in terms of statistical analysis and outcome measures, some articles did not provide clear mean and SD values before and after treatment, which could further contribute to heterogeneity in the meta-analysis results. Our study would greatly benefit from a more comprehensive and precise analysis if more studies on BoNT-A for fasciitis become available in the future.

## 6. Conclusion

In summary, BoNT-A had significant therapeutic effects on the improvement of fasciitis at 3 specific sites, with the strongest pain improvement observed in plantar fasciitis, followed by neck shoulder and low back fasciitis. The potential mechanism of action of BoNT-A suggests it may be a powerful treatment for alleviating the interplay between fascia, muscles, and pain. However, more research is needed to evaluate the long-term benefits and potential risks associated with repeated BTX treatments.

## Acknowledgments

We thank all the participants of this study and the professors who provided intellectual advisors for this study.

## Author contributions

**Conceptualization:** Tong-Tong Li, Zhong-Yuan Liu, Zhi-Wen Zhang.

**Data curation:** Tong-Tong Li, Zhong-Yuan Liu.

**Formal analysis:** Zhi-Wen Zhang.

**Investigation:** Zhi-Wen Zhang, Ling Xiong.

**Methodology:** Tong-Tong Li, Zhong-Yuan Liu, Zhi-Wen Zhang.

**Supervision:** Ling Xiong, Zhi-Wen Zhang.

**Validation:** Ling Xiong.

**Writing – original draft:** Tong-Tong Li, Zhong-Yuan Liu.

**Writing – review & editing:** Tong-Tong Li.

## Supplementary Material




