# Supplementary material for: Clinical efficacy of botulinum toxin type A in the treatment of fasciitis pain: A systematic review and meta-analysis
Source: Medicine (Baltimore). 2023 Jul 28;102(30):e34461. doi: 10.1097/MD.0000000000034461 (PMC10378743; doi:10.1097/MD.0000000000034461)
Supplement: Supplementary file 1 [file medi-102-e34461-s001.pdf]

Pubmed：[All Fields](fasciitis OR fasciopathy OR chronic fasciitis OR lumbar back fasciitis OR neck and shoulder fasciitis OR Plantar fasciitis OR inflammation pain) AND (Botulinum toxin type A OR BoNTA OR BTX OR BoNT)

Embase：(('fasciitis'/exp OR fasciitis OR fasciopathy OR 'chronic fasciitis' OR (chronic AND ('fasciitis'/exp OR fasciitis)) OR 'lumbar back fasciitis' OR (lumbar AND ('back'/exp OR back) AND ('fasciitis'/exp OR fasciitis)) OR 'neck'/exp OR neck) AND ('shoulder fasciitis' OR (('shoulder'/exp OR shoulder) AND ('fasciitis'/exp OR fasciitis))) OR 'plantar fasciitis'/exp OR 'plantar fasciitis' OR (plantar AND ('fasciitis'/exp OR fasciitis)) OR 'inflammation pain' OR (('inflammation'/exp OR inflammation) AND ('pain'/exp OR pain))) AND ('botulinum toxin type a'/exp OR 'botulinum toxin type a' OR (botulinum AND ('toxin'/exp OR toxin) AND type AND a) OR bonta OR 'btx'/exp OR btx OR bont)

Cochrane Library：[All text](fasciitis OR fasciopathy OR chronic fasciitis OR lumbar back fasciitis OR neck and shoulder fasciitis OR Plantar fasciitis OR inflammation pain) AND (Botulinum toxin type A OR BoNTA OR BTX OR BoNT)

Web of Science：(fasciitis OR fasciopathy OR chronic fasciitis OR lumbar back fasciitis OR neck and shoulder fasciitis OR Plantar fasciitis OR inflammation pain) AND (Botulinum toxin type A OR BoNTA OR BTX OR BoNT)
